# Uncoupling of energy metabolism and myocardial function in early left atrial dysfunction: a metabolomics study

**DOI:** 10.3389/fcvm.2026.1817236

**Published:** 2026-07-21

**Authors:** Haiying Jia, Dong An, Hui Yang, Jiajie Mei, Yan Wang, Jiabin Wen, Liming Xu, Lili Yin

**Affiliations:** 1 International Medical Department, The Second Affiliated Hospital of Dalian Medical University, Dalian, China; 2 Scientific Research Center, The Second Affiliated Hospital of Dalian Medical University, Dalian, China; 3 Department of Cardiology, The Second Affiliated Hospital of Dalian Medical University, Dalian, China

**Keywords:** energy metabolism, heart failure, left atrial dysfunction, machine learning, metabolomics

## Abstract

**Background:**

Left atrial (LA) dysfunction is a key preclinical phenotype of heart failure (HF), closely linked to HF progression, yet its molecular mechanisms remain unclear. Energy metabolic abnormalities may critically influence early LA dysfunction. This study aimed to explore the metabolic mechanisms underlying early LA dysfunction and develop a predictive model using metabolomics.

**Methods:**

We enrolled 315 participants, classified into early LA dysfunction and healthy control groups based on LA reservoir strain assessed via cardiovascular magnetic resonance (CMR). Targeted metabolomics was applied to detect plasma metabolites, and machine learning methods were used to construct predictive models. In addition, metabolic pathway and mediation analyses were also performed.

**Results:**

A total of 11 metabolites were significantly altered in early LA dysfunction, including elevated short-chain acylcarnitines (C4OH, C5:1) and proline, and decreased linoleic acid. C4OH and C5:1 correlated positively, while linoleic acid negatively correlated with LA reservoir strain. These metabolites distinguished early LA dysfunction from controls effectively (AUC=0.716; 95% CI: 0.616–0.817). Mediation analysis indicated that C5:1 and C5OH exacerbated LA dysfunction partly via obesity (BMI).

**Conclusions:**

Energy metabolic-mechanical function uncoupling may underlie early LA dysfunction. C4OH, C5:1, linoleic acid, and proline represent potential biomarkers and candidate metabolic pathways for future therapeutic investigation for early HF detection and intervention.

**Registration number:**

ChiCTR2200057991; Date of registration: 2022-03-25. URL: http://www.chictr.org.cn/showproj.aspx?proj=162316.

## 1 Introduction

Heart failure (HF) represents a major global health burden characterized by high prevalence, mortality rates, and healthcare costs (1). Early detection during the preclinical phase is clinically significant for preventing disease progression to symptomatic stages. However, current screening strategies for asymptomatic individuals exhibit substantial evidence gaps, highlighting the urgent need to identify reliable biomarkers or predictive models capable of detecting preclinical HF manifestations.

Left atrial (LA) dysfunction represents a critical phenotypic manifestation during the preclinical phase of HF, demonstrating a causal relationship with HF development and progression (2). Early-stage LA dysfunction is characterized by preserved structural parameters but impaired myocardial strain, with reduced LA reservoir strain serving as a validated marker of preclinical HF (3). Notably, this early LA dysfunction (manifested by diminished LA reservoir strain) not only predicts subsequent HF onset but also correlates significantly with worsening New York Heart Association (NYHA) functional classification (4). These observations underscore the importance of elucidating the molecular mechanisms underlying LA dysfunction to facilitate precise diagnosis and early intervention in preclinical HF.

In recent years, interests have been grown in the relationship between atrial metabolic remodeling and LA dysfunction. Accumulating evidences indicated that disturbances in energy metabolism constituted a key determinant of impaired LA function (5). Preclinical studies demonstrated that metabolic risk factors such as obesity might compromise LA performance through mitochondrial dysfunction and lipotoxic pathways (6), although the precise molecular mechanisms remained incompletely understood. Of particular relevance there was tight coupling between cardiomyocyte energy metabolism and contractile function suggesting that metabolic perturbations might directly influence LA function (7). Since circulating metabolites which represent the end products of cellular processes, integrate genetic predisposition, environmental exposures, and lifestyle factors, it has been emerged as an efficient, standardized, and scalable research strategy for investigating early-stage HF (8). Oexner et al. recently demonstrated that alterations in tricarboxylic acid cycle intermediates in serum metabolomic profiles effectively predicted HF incidence in healthy populations (9). These developments position metabolomics as an innovative paradigm for elucidating the underlying molecular mechanisms of LA dysfunction during the preclinical phase of HF.

Therefore, this study aims to investigate the molecular mechanisms of early-stage LA dysfunction by integrating metabolomics with machine learning approaches. A predictive model based on metabolic signatures will be established to identify preclinical HF, thereby to provide new scientific evidence for early HF screening and intervention strategies.

## 2 Methods

### 2.1 Research subjects and groupings

According to the diagnostic criteria published by European Journal of Heart Failure in 2022, the patients with strain index for left atrial reserve (LAEs) less than 23% were included in the LA reserve dysfunction group, while the patients with LAEs ≥ 23% were included in the normal group (10). Patients with atrial fibrillation or significant valvular disease, including mitral regurgitation, were excluded, so that the cohort reflected early, subclinical LA reservoir dysfunction independent of these established causes of atrial impairment. All participants provided written informed consent after being fully briefed on the study's purpose and procedures. This study was approved by the Ethics Committee of the Second Affiliated Hospital of Dalian Medical University (Approval No. 17, 2023) and adhered to the ethical guidelines of the Declaration of Helsinki.

### 2.2 Research methodology

Each participant received a comprehensive medical evaluation, which included a detailed clinical history, physical examination, and imaging tests. On the morning of the cardiovascular magnetic resonance (CMR) examination, participants were required to fast for at least 8 h. Venous blood samples were collected by a trained nurse following a standardized procedure, using ethylenediaminetetraacetic acid (EDTA)-containing vacuum blood collection tubes. The samples were stored at room temperature and processed within 6 h. All blood samples were handled by laboratory physicians, with the exception of those for targeted metabolic profiling tests.

CMR imaging was employed as the standard method for assessing the morphology and function of the LA. Imaging was performed by two experienced radiologists from the Second Affiliated Hospital of Dalian Medical University. To preserve the single-blind design of the study, all personally identifiable informations, such as age, gender, and hospitalization number, were excluded from the image analysis process. Tissue tracking technology was utilized to monitor myocardial voxels in the long-axis sections of the horizontal four-chamber and vertical two-chamber views. Key parameters included LAEs, LAmax (left atrial maximum strain), LVEF (left ventricular ejection fraction) and LV-EDV (left ventricular end-diastolic volume index) were measured. For consistency and reliability, strain index values were derived from the average of three independent measurements.

Targeted metabolic profiling was conducted by sending the blood samples to the Dalian Institute of Chemical Physics, Chinese Academy of Sciences, for quantitative analysis using high-performance liquid chromatography-tandem mass spectrometry (LC-MS/MS). Pathway enrichment was based on the KEGG PATHWAY database (KEGG accessed Feb. 10, 2018; Kanehisa et al.).

### 2.3 Predictive model construction

Given the significant imbalance between positive and negative diagnostic events, propensity score matching (PSM) was performed using the ‘MatchIt’ package in R software (version 4.3.2) to balance the confounding factors between the study groups at a ratio of 1:6. This process ultimately identified 315 patients as the study subjects. Normally distributed continuous variables were expressed as mean ± standard deviation ($\bar{x}$±SD). These group comparisons were conducted by using an independent samples t-test. Categorical variables were presented as frequencies and percentages. These group comparisons were conducted by using the chi-square test.

Univariate logistic regression analysis was subsequently performed on the contents of small molecule metabolites. To avoid excluding important factors, variables with *P*-values less than 0.1 were preliminarily screened, followed by Lasso (least absolute shrinkage and selection operator) regression using the `glmnet` package in R. Lasso regression incorporated a penalty function to continuously shrink the coefficients, in order to reduce model overfitting and address multicollinearity, thus efficiently select variables. A 10-fold cross-validation approach was employed to determine the optimal penalty term (*λ*). To retain important features, the minimum *λ* value was chosen.

In this study, 75% of the dataset was designated as the training set, while the remaining 25% allocated as the validation set. To ensure the reliability and rigor of the results, only variables with *P*-value <0.05 were included in the analysis. A forest plot was generated using the `forestplot` package in R. Additionally, the `RMS` package was employed to construct a nomogram based on the results, to integrate the predictors and assign score values in order to calculate the total score and estimate the probability of left atrial reserve dysfunction for each patient. To assess the predictive power of the nomogram, the area under the curve (AUC) was calculated. The receiver operating characteristic (ROC) curve was plotted to evaluate the accuracy and precision of the metabolic model on identifying patients with left atrial reserve dysfunction. The model's fit was further assessed using the Hosmer-Lemeshow test, with a *P*-value > 0.05 indicating a good model fit.

### 2.4 Mediation analysis

Mediation analysis was conducted using SPSS 27, with the `PROCESS` plugin installed and utilized. Through model 4 and the Bootstrap method, we examined the mediating role of small molecule metabolites in the relationship between BMI and left atrial reserve function, and set age and gender as control variables. The effect size, *β* value, and 95% confidence interval (CI) of the mediation effect by resampling 5,000 times were calculated through Bootstrap method. A positive correlation was evaluated based on whether the *β* value was greater than 0. If the 95% CI of an indirect effect did not include 0, the mediating effect was considered statistically significant. For small molecule metabolites that showed significant mediating effects in the simple mediation analysis, we further applied model 6 for chain mediation analysis to explore potential associations between different metabolites. A statistical significance was indicated as *p* < 0.05.

## 3 Results

### 3.1 Baseline characteristics of the study subjects

A total of 315 patients were included in this study (Table 1). During comparing age and BMI between the two groups, we found that the LA reserve dysfunction group (57.20 ± 12.45 years) was older than the normal group (53.80 ± 9.83 years) and had a higher BMI (26.94 ± 3.41 kg/m vs. 25.53 ± 3.51 kg/m ). There was no statistically significant difference in ventricular function or structural characteristics (E/A, E/e’, LVEF, LV-EDV) between the two groups.

**Table 1:** Comparison of baseline features in the LA reserve dysfunction group with the normal group.

Variable	Category	LA reserve dysfunction group	Normal group	*P*-value
Number		45	270	
Age		57.20 ± 12.45	53.80 ± 9.83	0.04
BMI (kg/m )		26.94 ± 3.41	25.53 ± 3.51	0.017
Gender (%)	female	14 (31.1)	125 (46.3)	0.058
	male	31 (68.9)	145 (53.7)	
BP (%)	no	20 (44.4)	137 (50.7)	0.434
	yes	25 (55.6)	133 (49.3)	
Diabetes (%)	no	26 (57.8)	167 (61.9)	0.603
	yes	19 (42.2)	103 (38.1)	
Smoking (%)	no	35 (77.8)	218 (80.7)	0.643
	yes	10 (22.2)	52 (19.3)	
Drink (%)	no	39 (86.7)	248 (91.9)	0.258
	yes	6 (13.3)	22 (8.1)	
HbA1c (%)		5.70 ± 0.93	5.89 ± 0.99	0.445
FBG (mmol/L)		6.09 ± 2.05	5.65 ± 1.62	0.107
LDLC (mmol/L)		2.91 ± 1.11	3.02 ± 0.84	0.458
HDLC (mmol/L)		1.21 ± 0.27	1.25 ± 0.31	0.342
TC (mmol/L)		4.86 ± 1.24	5.13 ± 1.01	0.114
RC (mmol/L)		0.73 ± 0.32	0.85 ± 0.42	0.076
TG (mmol/L)		1.62 ± 0.93	1.76 ± 1.29	0.485
Urea (mmol/L)		5.86 ± 1.37	5.67 ± 1.29	0.351
UA (umol/L)		399.09 ± 120.72	367.59 ± 99.23	0.057
HCY (umol/L)		11.54 ± 5.35	11.04 ± 4.89	0.536
ApoA1 (g/L)		1.50 ± 0.23	1.54 ± 0.26	0.342
ApoB (g/L)		0.89 ± 0.25	0.91 ± 0.22	0.701
HsCRP (mg/L)		1.55 ± 3.42	2.19 ± 5.72	0.47
eGFR (ml/min)		109.73 ± 32.23	116.35 ± 31.75	0.207
BNP(pg/mL)		22.1 ± 17.47	19.14 ± 16.6	0.326
Cardiac ultrasound				
E/A		0.91 ± 0.33	0.94 ± 0.31	0.489
E/e’		8.7 ± 2.4	8.1 ± 2.13	0.192
CMR				
LAEs		19.29 ± 4.56	39.6 ± 11.24	<0.001
LAmax		64 ± 26.65	58.21 ± 19.60	0.169
LVEF		61.08 ± 5.94	61.21 ± 5.79	0.895
LV-EDV		132.14 ± 33.18	134.12 ± 27.22	0.705

### 3.2 Characteristics of the plasma metabolic profiles of the study subjects

Over the 58 small molecule metabolites analyzed, 11 were found to be significantly different (*P* < 0.1) between the left atrial reserve dysfunction group and the control group as shown in Table 2. These included four fatty acids (*ω*-6 fatty acids, T-PUFA, linoleic acid and arachidonic acid), six acylcarnitines (C4, C4OH, C5OH, C5:1, C12 and C22) and one amino acid (proline). Specifically, *ω*-6 fatty acids, T-PUFA, linoleic acid and arachidonic acid were negatively correlated with LA reserve function, while C4, C4OH, C5OH, C5:1, C12, C22 and proline were positively correlated with LA reserve function. Additionally, participants were divided into obese and non-obese groups based on a BMI threshold of 25. In the obese group (Table 2), the differences of small molecule metabolites in LA reserve function were more pronounced. Specifically, linoleic acid was identified as a protective metabolite in the obese group, while C2, C4, C4OH, C5OH, C5:1, C14DC, C14:1 and C22 were identified as detrimental metabolites. In the non-obese group, DHA, C0, C3, and C4DC were protective, while C4OH, C12 and proline were associated with a higher risk.

**Table 2:** Univariate analysis of metabolites in all participants, obese patients, and non-obese patients.

Variable	All participants (*n* = 315)			Obese patients (*n* = 174)			Non-obese patients (*n* = 141)		
	OR	95%*CI*	*P*	OR	95%*CI*	*P*	OR	95%*CI*	*P*
*ω*-3 Fatty acids	0.751	(0.499,1.131)	0.171	0.765	(0.469,1.247)	0.282	0.487	(0.205,1.157)	0.103
ω-6 Fatty acids	0.697	(0.477,1.019)	0.063	0.72	(0.453,1.146)	0.166	0.568	(0.286,1.126)	0.105
TFA	0.809	(0.566,1.157)	0.246	0.792	(0.509,1.233)	0.301	0.679	(0.344,1.341)	0.265
SFA	1.012	(0.74,1.386)	0.938	0.982	(0.666,1.448)	0.928	0.874	(0.478,1.597)	0.661
T-MUFA	0.874	(0.615,1.242)	0.452	0.752	(0.466,1.213)	0.242	0.936	(0.513,1.707)	0.829
T-PUFA	0.679	(0.459,1.004)	0.052	0.679	(0.42,1.097)	0.114	0.557	(0.263,1.182)	0.128
ALA	0.817	(0.562,1.186)	0.287	0.734	(0.446,1.207)	0.223	0.846	(0.447,1.6)	0.607
EPA	0.853	(0.6,1.213)	0.377	0.834	(0.544,1.28)	0.406	0.657	(0.309,1.396)	0.274
DHA	0.767	(0.546,1.077)	0.126	0.826	(0.555,1.228)	0.344	0.487	(0.219,1.084)	0.078
DPA	0.721	(0.473,1.099)	0.129	0.729	(0.441,1.204)	0.217	0.561	(0.236,1.334)	0.191
Linoleic acid	0.721	(0.522,0.994)	0.046	0.698	(0.473,1.032)	0.072	0.682	(0.376,1.237)	0.208
Arachidonic acid	0.701	(0.496,0.991)	0.044	0.765	(0.507,1.154)	0.202	0.591	(0.306,1.142)	0.118
C0	1.039	(0.759,1.422)	0.81	1.259	(0.864,1.835)	0.23	0.539	(0.271,1.075)	0.08
C2	1.176	(0.874,1.583)	0.284	1.418	(0.991,2.029)	0.056	0.691	(0.351,1.362)	0.286
C3	1.104	(0.817,1.492)	0.521	1.208	(0.841,1.735)	0.306	0.488	(0.211,1.131)	0.094
C4	1.287	(0.968,1.712)	0.082	1.464	(1.038,2.064)	0.037	0.874	(0.459,1.662)	0.681
C4OH	1.531	(1.172,2)	0.002	1.436	(1.044,1.975)	0.03	1.525	(0.99,2.35)	0.056
C4DC	0.923	(0.667,1.275)	0.626	1.043	(0.713,1.526)	0.828	0.555	(0.281,1.099)	0.091
C5	1.076	(0.79,1.466)	0.643	1.091	(0.745,1.598)	0.654	0.803	(0.415,1.557)	0.517
C5OH	1.497	(1.131,1.98)	0.005	1.664	(1.164,2.381)	0.005	0.928	(0.51,1.689)	0.808
C5DC	0.9	(0.646,1.253)	0.532	0.783	(0.522,1.173)	0.235	1.108	(0.652,1.882)	0.705
C5:1	1.427	(1.061,1.918)	0.019	1.612	(1.115,2.33)	0.011	0.905	(0.501,1.633)	0.74
C6	1.081	(0.817,1.429)	0.587	1.007	(0.688,1.473)	0.97	1.266	(0.734,2.184)	0.396
C6DC	1.03	(0.753,1.409)	0.854	1.175	(0.808,1.707)	0.398	0.696	(0.364,1.333)	0.274
C8	1.035	(0.784,1.366)	0.809	0.936	(0.572,1.531)	0.792	1.384	(0.886,2.163)	0.154
C10	1.07	(0.813,1.41)	0.629	1.006	(0.688,1.471)	0.976	1.278	(0.78,2.093)	0.33
C12	1.314	(0.982,1.758)	0.066	1.081	(0.743,1.573)	0.684	1.785	(1.092,2.917)	0.021
C14	0.963	(0.679,1.365)	0.831	1.022	(0.696,1.498)	0.913	0.857	(0.386,1.982)	0.748
C14OH	1.07	(0.787,1.454)	0.666	1.08	(0.742,1.571)	0.689	1.044	(0.598,1.824)	0.879
C14DC	1.181	(0.917,1.521)	0.198	1.582	(1.095,2.287)	0.015	1.068	(0.669,1.705)	0.782
C14:1	1.149	(0.902,1.463)	0.261	1.446	(1.018,2.054)	0.039	1.104	(0.727,1.676)	0.643
C16	1.114	(0.827,1.5)	0.477	1.341	(0.93,1.934)	0.116	0.7	(0.346,1.417)	0.321
C16OH	1.074	(0.787,1.467)	0.653	1.066	(0.729,1.558)	0.742	1.043	(0.591,1.839)	0.886
C16:1-OH	0.947	(0.663,1.352)	0.764	0.997	(0.678,1.465)	0.988	0.843	(0.358,1.981)	0.695
C18	1.153	(0.847,1.568)	0.365	1.25	(0.86,1.818)	0.242	0.992	(0.559,1.761)	0.979
C20	1.049	(0.768,1.432)	0.766	1.309	(0.91,1.883)	0.147	0.654	(0.336，1.273)	0.212
C22	1.273	(0.99,1.637)	0.06	1.333	(0.954,1.862)	0.092	1.004	(0.568,1.776)	0.989
C24	0.868	(0.606,1.243)	0.439	0.962	(0.652,1.421)	0.847	0.599	(0.25,1.439)	0.252
C26	0.968	(0.694,1.351)	0.849	1.184	(0.828,1.692)	0.355	0.451	(0.136,1.492)	0.192
Valine	1.065	(0.775,1.461)	0.699	0.939	(0.639,1.378)	0.747	0.993	(0.561,1.759)	0.981
Leucine	1.14	(0.837,1.555)	0.406	0.888	(0.601,1.313)	0.553	1.367	(0.804,2.327)	0.249
Histidine	1.033	(0.76,1.403)	0.838	1.02	(0.7,1.487)	0.917	1.082	(0.626,1.87)	0.778
Lysine	1.152	(0.845,1.57)	0.371	1.084	(0.74,1.587)	0.679	1.313	(0.763,2.259)	0.326
Methionine	0.985	(0.717,1.353)	0.926	0.872	(0.584,1.303)	0.505	1.134	(0.658,1.955)	0.651
Phenylalanine	0.937	(0.679,1.294)	0.693	0.838	(0.558,1.258)	0.394	0.899	(0.498,1.624)	0.724
Threonine	0.793	(0.564,1.117)	0.185	0.72	(0.468,1.108)	0.135	0.913	(0.508,1.641)	0.762
Tryptophan	0.922	(0.67,1.269)	0.619	0.916	(0.621,1.351)	0.659	0.856	(0.476,1.54)	0.604
Alanine	1.055	(0.771,1.443)	0.737	0.912	(0.617,1.35)	0.647	1.127	(0.644,1.974)	0.675
Arginine	1.027	(0.75,1.405)	0.869	1.05	(0.719,1.535)	0.8	0.883	(0.488,1.598)	0.681
Asparagine	1.226	(0.905,1.661)	0.188	1.073	(0.733,1.57)	0.719	1.228	(0.722,2.088)	0.448
Aspartic acid	1.145	(0.841,1.56)	0.389	1.113	(0.764,1.623)	0.577	1.044	(0.592,1.842)	0.88
Cysteine	1.179	(0.879,1.581)	0.273	1.15	(0.793,1.699)	0.461	1.295	(0.795,2.11)	0.299
Glutamine	1.037	(0.758,1.418)	0.821	0.962	(0.653,1.417)	0.843	1.218	(0.711,2.088)	0.473
Glutamic acid	1.095	(0.808,1.483)	0.558	1.101	(0.759,1.595)	0.613	1.109	(0.647,1.903)	0.706
Glycine	0.893	(0.641,1.244)	0.502	0.867	(0.58,1.296)	0.486	1.122	(0.651,1.933)	0.679
Proline	1.559	(1.159,2.096)	0.003	1.269	(0.884,1.821)	0.197	2.134	(1.192,3.821)	0.011
Serine	1.024	(0.748,1.402)	0.881	1.012	(0.69,1.485)	0.951	1.006	(0.568,1.783)	0.983
Tyrosine	0.927	(0.672,1.279)	0.645	0.821	(0.55,1.227)	0.336	0.938	(0.525,1.675)	0.829

### 3.3 A prediction model for LA reserve dysfunction based on targeted metabolomics

**Figure 1:**
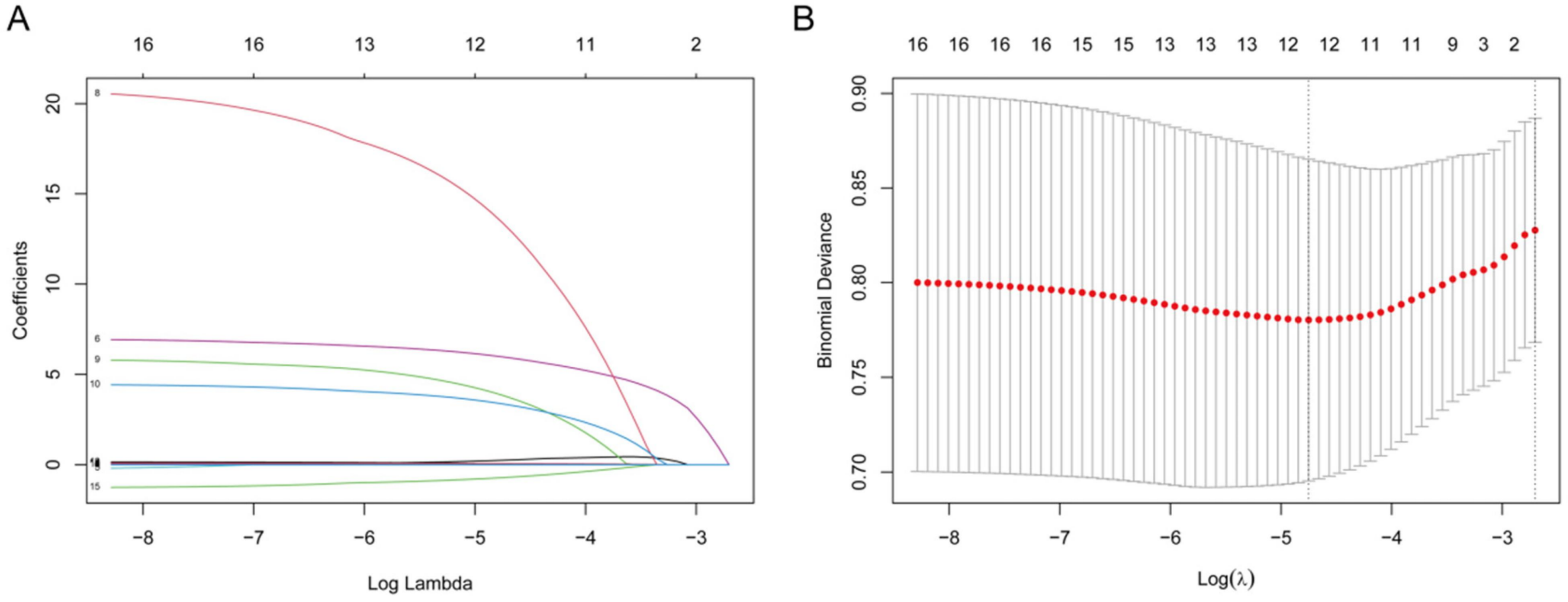
Model variable screening based on lasso regression. **(A)** Lasso coefficient distribution for 17 variables; **(B)** Lasso the cross-validation results. The dotted line on the left indicated the value of the harmonic parameter log (*λ*) when the model error is minimized.

The Lasso regression method was employed to analyze the statistically significant variables from the patients’ general profiles (Table 1) and the differential metabolic profiles (Table 2). The lambda min value was determined to select the variables (as shown in Figure 1). After screening, when log(*λ*) = 0.008, 12 predictors were identified, including one fatty acid (linoleic acid), five acylcarnitines (C4OH, C5OH, C5:1, C12, C22), one amino acid (proline), age, sex, BMI, UA and RC.

**Figure 2:**
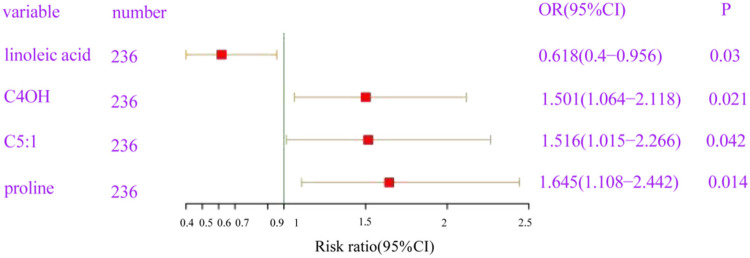
Multivariate stepwise logistic regression of prediction model in forest plot. The final model incorporated linoleic acid, C5:1, C4OH, and proline.

**Figure 3:**
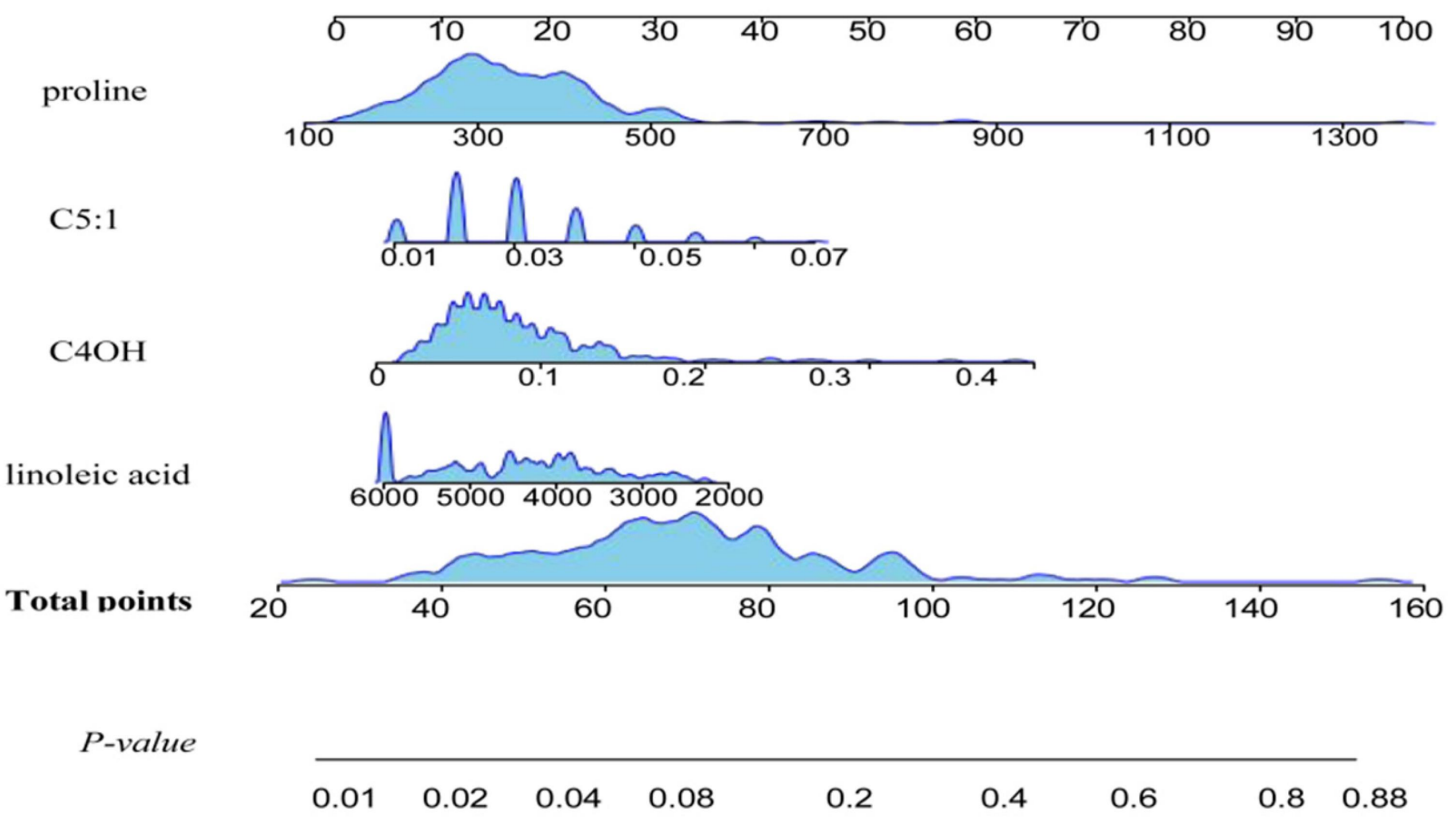
Nomogram of four independent predictors based on logistics regression. The nomogram displays the score allocated to each independent risk factor.

A prediction model for LA reserve dysfunction was constructed using multivariate stepwise logistic regression (Figure 2). The final model incorporated linoleic acid, C5:1, C4OH, and proline (*P* < 0.05). Specifically, linoleic acid (OR = 0.618, 95% CI: 0.4–0.956) was identified as a protective factor, while C5:1 (OR = 1.516, 95% CI: 1.015–2.266), C4OH (OR = 1.501, 95% CI: 1.064–2.118) and proline (OR = 1.645, 95% CI: 1.108–2.442) were recognized as risk factors. Based on this model, a nomogram was constructed (Figure 3) to predict the individual risk of LA reserve dysfunction.

### 3.4 Model training and validation

**Figure 4:**
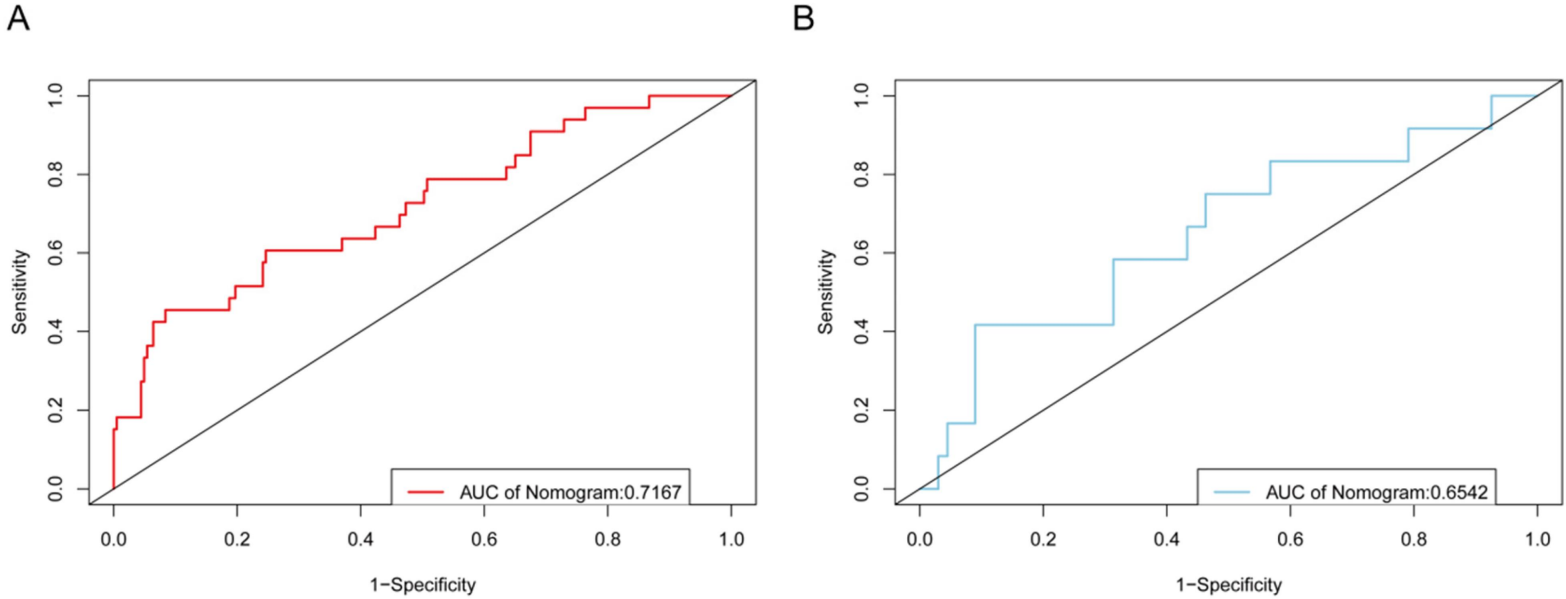
The ROC curves of the prediction models. (**A**) ROC of the training set in the LA reserve dysfunction model. (**B**) the validation set in the LA reserve dysfunction model.

The discriminatory power of the LA function risk model was assessed using the ROC curve (Figure 4). In the training set, the AUC of the model was 0.716, with a 95% CI ranging from 0.616 to 0.817. In the independent validation set, the AUC was 0.654, with a 95% CI between 0.472 and 0.835. Finally, the *P* value from the Hosmer-Lemeshow test was greater than 0.05 in both the training and validation sets. These results indicated that the model we developed was reliable.

### 3.5 The mediating role of small molecule metabolites in the relationship between BMI and LA reserve function

In the results of a one-way analysis of the metabolic profile related to LA reserve function (Table 2), our subgroup analysis indicated that small molecules had different effects on LAEs based on BMI status. Building on these findings, we explored the mediating role of various small molecule metabolites in the relationship between BMI and LAEs through simple mediation analysis (Table 3). If C5OH was used as a mediating variable, the indirect effect was −0.043 (95% CI: −0.079–−0.016). Similarly, if C5:1 was used as a mediating variable, the indirect effect was −0.016 (95% CI: −0.039–−0.001). These results demonstrated that both C5OH and C5:1 played mediating roles in the relationship between BMI and LAEs after the variables age and gender were controlled.

**Table 3:** The simple mediating analysis of metabolites.

Variable	Effect value	OR	Lower of 95% CI	Upper of 95% CI
ω-3 Fatty acids	−0.001	0.007	−0.016	0.013
ω-6 Fatty acids	0.002	0.005	−0.006	0.014
TFA	−0.002	0.008	−0.018	0.014
SFA	−0.007	0.010	−0.028	0.014
T-MUFA	−0.017	0.011	−0.039	0.003
T-PUFA	0.004	0.006	−0.006	0.018
ALA	−0.008	0.007	−0.024	0.005
EPA	−0.008	0.007	−0.025	0.005
DHA	0.000	0.005	−0.010	0.010
DPA	0.001	0.004	−0.007	0.011
Linoleic acid	0.003	0.006	−0.008	0.018
Arachidonic acid	−0.004	0.006	−0.019	0.006
C0	−0.001	0.006	−0.014	0.011
C2	0.002	0.007	−0.012	0.016
C3	−0.012	0.017	−0.047	0.019
C4	−0.009	0.009	−0.032	0.003
C4OH	−0.017	0.011	−0.041	0.003
C4DC	0.005	0.008	−0.009	0.024
C5	−0.024	0.015	−0.057	0.002
C5OH	−0.043	0.016	−0.079	−0.016
C5DC	0.002	0.005	−0.009	0.012
C5:1	−0.016	0.010	−0.039	−0.001
C6	−0.003	0.006	−0.015	0.009
C6DC	−0.002	0.006	−0.017	0.009
C8	0.002	0.007	−0.012	0.020
C10	0.000	0.005	−0.007	0.015
C12	−0.002	0.005	−0.014	0.006
C14	0.000	0.003	−0.009	0.005
C14OH	−0.005	0.006	−0.019	0.005
C14DC	0.005	0.008	−0.012	0.019
C14:1	−0.001	0.007	−0.024	0.002
C16	−0.007	0.007	−0.024	0.002
C16OH	−0.003	0.006	−0.016	0.008
C16:1-OH	−0.001	0.006	−0.020	0.002
C18	−0.004	0.005	−0.016	0.003
C20	0.000	0.004	−0.006	0.009
C22	−0.006	0.008	−0.021	0.009
C24	−0.004	0.007	−0.020	0.009
C26	−0.002	0.005	−0.016	0.004
Valine	−0.021	0.016	−0.051	0.010
Leucine	−0.042	0.022	−0.087	0.003
Histidine	−0.008	0.007	−0.026	0.002
Lysine	0.001	0.004	−0.007	0.012
Methionine	0.000	0.006	−0.011	0.012
Phenylalanine	−0.006	0.014	−0.035	0.022
Threonine	0.000	0.003	−0.008	0.006
Tryptophan	0.000	0.003	−0.008	0.006
Alanine	−0.011	0.014	−0.041	0.015
Arginine	−0.001	0.004	−0.010	0.008
Asparagine	−0.007	0.020	−0.046	0.032
Aspartic acid	−0.012	0.010	−0.034	0.005
Cysteine	0.003	0.005	−0.005	0.016
Glutamine	0.000	0.003	−0.008	0.007
Glutamic acid	−0.001	0.004	−0.011	0.006
Glycine	−0.004	0.012	−0.027	0.019
Proline	−0.007	0.007	−0.023	0.007
Serine	−0.003	0.005	−0.014	0.008
Tyrosine	−0.017	0.015	−0.049	0.012

**Figure 5:**
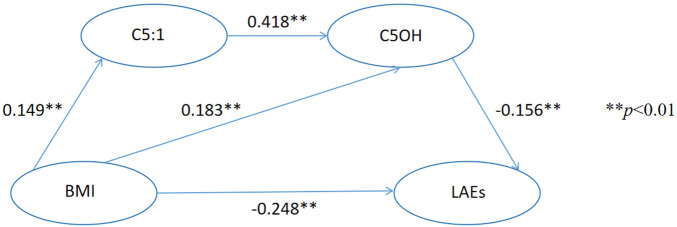
The mediating role of C5:1,C5OH in BMI and LAEs. Chain mediation modelling demonstrated positive BMI-metabolite relationships (C5:1: *β*=0.149; C5OH: *β*=0.183) alongside negative associations with LAEs (*β*=−0.248).

Since both C5:1 and C5OH are short-chain acylcarnitines, a chain mediation analysis was further performed (Table 4). After adjusting for age and gender, we found that the total effect of BMI on LAEs was −0.045 (95% CI: −0.079–−0.015), with a direct effect of −0.293 (95% CI: −0.399–−0.188), which was statistically significant. When C5OH was considered as a mediating variable, the indirect effect was −0.029 (95% CI: −0.058–−0.006). When both C5:1 and C5OH were included as mediating variables, the combined indirect effect was −0.01 (95% CI: −0.024–−0.001). Based on the *β* values, we confirmed the relationships between BMI, C5:1, C5OH, and LAEs (Figure 5). Specifically, BMI was positively correlated with both C5:1 and C5OH, but negatively correlated with LAEs (*P*-value < 0.01). These findings suggested that the relationship between BMI and LAEs might be partially mediated by the short-chain acylcarnitines C5:1 and C5OH.

**Table 4:** C5:1 and C5OH mediated chain mediation analysis between BMI and LAEs.

Model pathways	Effect	OR	Lower of 95% CI	Upper of 95% CI	Percentage of total effect
BMI→LAEs	−0.293	0.054	−0.399	−0.188	
BMI→C5:1→LAEs	−0.007	0.009	−0.027	0.01	
BMI→C5OH→LAEs	−0.029	0.014	−0.058	−0.006	63.70%
BMI→C5:1→C5OH→LAEs	−0.01	0.006	−0.024	−0.001	21.70%
Total effect	−0.045	0.016	−0.079	−0.015	100%
Direct effect	−0.248	0.055	−0.356	−0.141	

## 4 Discussion

This study employed an integrated metabolomics and machine learning approach to systematically investigate the molecular mechanisms of early LA dysfunction and successfully developed a predictive model based on small-molecule metabolites. Our work provided the first evidence to demonstrate significant associations between specific metabolites (including acylcarnitines, linoleic acid, and proline) and early LA dysfunction (defined by significantly reduced LA reservoir strain).

In our preliminary study, we employed a quasi-targeted metabolomics approach to characterize small-molecule metabolite profiles in patients with early LA dysfunction. Among 661 metabolites analyzed, 141 showed differential expression based on predefined thresholds (fold change >1.2 or <0.833; *P* < 0.05) (Supplementary Figure S1). Compared with controls, the LA reservoir dysfunction group exhibited 140 upregulated metabolites and 1 downregulated metabolite. These findings demonstrate significant metabolic perturbations in early LA dysfunction, providing a strong rationale for employing targeted metabolomics to elucidate the molecular mechanisms underlying LA dysfunction. KEGG pathway analysis from our preliminary research identified significant associations between early LA dysfunction which was quantified by reduced LA reservoir strain, and dysregulated energy metabolism pathways, particularly ubiquinone and other terpenoid quinone biosynthesis (Supplementary Figure S2) (11–13). The ubiquinone biosynthesis pathway generates essential cofactors and vitamin E (14), which may directly or indirectly disrupt myocardial energy homeostasis (15, 16). Studies showed that normal myocardial energy metabolism primarily relied on fatty acid β-oxidation. However, under pathological conditions, significant metabolic remodeling occurs, which could be characterized by reduced metabolic flexibility in the myocardium. This manifests as impaired fatty acid oxidation and a shift towards greater dependence on glucose and amino acid metabolism, ultimately leading to abnormal energy metabolism and myocardial dysfunction (17). Our targeted metabolomics data confirmed that impaired energy metabolism (including limited fatty acid β-oxidation and abnormal amino acid metabolism) was a key feature of early LA dysfunction. This supported the important finding that disrupted energy metabolism might represent a molecular mechanism of LA dysfunction (preclinical stage of heart failure).

Regarding fatty acid metabolism, this study identified two key acylcarnitines (C4OH and C5:1) and an unsaturated fatty acid (linoleic acid) as strongly associated with LA dysfunction in patients with early-stage impairment. A predictive model for LA dysfunction based on these small molecules demonstrated moderate diagnostic efficacy (AUC = 0.716, 95% CI: 0.616–0.817). Furthermore, mediation analysis revealed that two acylcarnitines (C5:1 and C5OH) not only may reflect metabolic alterations related to early LA dysfunction but also acted as mediators of obesity, potentially exacerbating early LA dysfunction. Study confirmed that obesity could induce mitochondrial fatty acid β-oxidation dysfunction, leading to acylcarnitine accumulation (18). Gao et al. reported that abnormal short-chain acylcarnitine levels might further impair LA conduit function and promote left ventricular injury, suggesting a direct impact of acylcarnitines on myocardial function (19). Additionally, Chen et al. demonstrated that elevated plasma C5OH levels could serve as a biomarker for cardiac systolic dysfunction in decompensated HF (20). Thus, C4OH and C5:1 were not only significant predictive biomarkers for LA dysfunction, but more importantly reflected the critical role of impaired energy metabolism (particularly limited fatty acid oxidation) in early myocardial dysfunction. These findings suggested a coupling between energy metabolism dysregulation and myocardial impairment, which might contribute to the progression of LA dysfunction.

Linoleic acid, an essential fatty acid, plays a critical role in regulating energy metabolism and protecting myocardial function. Reinders et al. demonstrated that low linoleic acid levels served as a biomarker for both reduced ventricular ejection function and left atrial enlargement in elderly populations (21). In our study, elevated linoleic acid levels not only reduced obesity-related effects but also showed a protective influence on early LA function. Early *in vitro* experiments showed that linoleic acid reduced fat accumulation and enhanced metabolic activity in mice, that effect attributed to the upregulation of mitochondrial uncoupling protein (UCP) and peroxisome proliferator-activated receptor alpha (PPAR*α*) activation (22). Furthermore, Maekawa et al. reported that linoleic acid constituted a key component of cardiolipin, a critical structural element in mitochondrial oxidative phosphorylation (OXPHOS). They suggested that linoleic acid supplementation improved mitochondrial dysfunction and alleviated cardiac impairment (23).

Regarding amino acid metabolism, we observed significantly elevated plasma proline levels in patients with early LA dysfunction. Diabetes is known to perturb polyunsaturated fatty-acid and amino-acid handling, including reductions in circulating linoleic acid and alterations in proline metabolism (24, 25). In the present cohort, however, the prevalence of diabetes, fasting glucose and HbA1c were comparable between groups (all *P* > 0.05; Table 1), and no glycaemic variable was retained in the LASSO-selected model. The metabolite differences we observed therefore arose against a background of similar glycaemic status, suggesting that they are unlikely to be driven primarily by diabetes in this sample. Aberrant proline metabolism may disrupt myocardial energy homeostasis through multiple pathways, potentially driving the uncoupling of energy metabolism from myocardial function. On the one hand, proline modulates of inflammatory responses via the PI3 K/AKT/NF-*κ*B signaling pathway (26); On the other hand, it acts as an energy metabolism regulator that exacerbates oxidative stress injury by depleting NAD(P)H and ATP (27). Notably, the dual role of proline may reflect the dynamic adaptation of cardiomyocytes to changing energy demands during disease progression. In early-stage HF, elevated proline levels could represent a compensatory mechanism that preserves the coupling between energy metabolism and myocardial contraction through mitochondrial regulation. However, as the disease advances, this adaptive response may become insufficient, ultimately leading to the decoupling of energy metabolism from cardiac function and establishing a vicious cycle. This proposed mechanism warrants further investigation through detailed mechanistic studies.

Our study had several limitations. We used BMI as the index of obesity because it is standardised, reproducible and available for all participants, facilitating comparison with prior literature. However, BMI cannot distinguish fat from lean mass or reflect fat distribution; measures such as waist circumference, body-composition analysis or epicardial adipose tissue quantification may capture obesity-related atrial risk more precisely and should be incorporated in future studies. The generalizability of the results may be limited because this study was an observational study of patients from northern China with a limited sample size and lack of validation by an external cohort. In addition, because of the observational design, the present study cannot determine whether altered circulating metabolites cause HF through LA dysfunction or contribute to HF progression through LA-independent systemic or myocardial pathways. Therefore, the identified metabolites should be interpreted as potential biomarkers and candidate mechanistic clues rather than validated therapeutic targets. Longitudinal studies and mechanistic experiments are needed to clarify causal relationships. Nevertheless, our work also provided important clues and insights for preclinical studies of heart failure, particularly the molecular mechanisms of early LA dysfunction and the potential value of early intervention. The candidate metabolites map onto defined enzymatic pathways of fatty-acid β-oxidation and acylcarnitine metabolism(e.g., CPT1/CPT2, ACADS, HADH), PPAR*α*-UCP-mediated linoleic-acid signalling, and proline metabolism (e.g., PRODH, PYCR1). In ongoing work we plan to integrate genetic approaches, including GWAS and Mendelian-randomisation analyses linking these metabolites to LA strain phenotypes, together with *in vitro* and *in vivo* functional validation.

In conclusion, this study suggested for the first time that “uncoupling of energy metabolism and myocardial function” might be the core molecular mechanism of early LA dysfunction and identified potential target molecules such as C4OH, C5:1, linoleic acid and proline. These metabolites were expected not only to serve as effective biomarkers for the diagnosis of early LA dysfunction, but also to provide novel targets for therapeutic intervention during the HF preclinical phase. These findings will provide significant theoretical supports for potential interventions to halt or slow down HF progression, while simultaneously guide future research priorities in both mechanistic understanding and clinical application.

## Data Availability

The original contributions presented in the study are included in the article/Supplementary Material, further inquiries can be directed to the corresponding authors.
