# Supplementary material for: Uncoupling of energy metabolism and myocardial function in early left atrial dysfunction: a metabolomics study
Source: Front Cardiovasc Med. 2026 Jul 21;13:1817236. doi: 10.3389/fcvm.2026.1817236 (PMC13435660; doi:10.3389/fcvm.2026.1817236)
Supplement: Supplementary file 1 [file Datasheet1.pdf]

Supplementary material

Figure S1. Volcanogram of differential metabolites in patients with LA reserve dysfunction

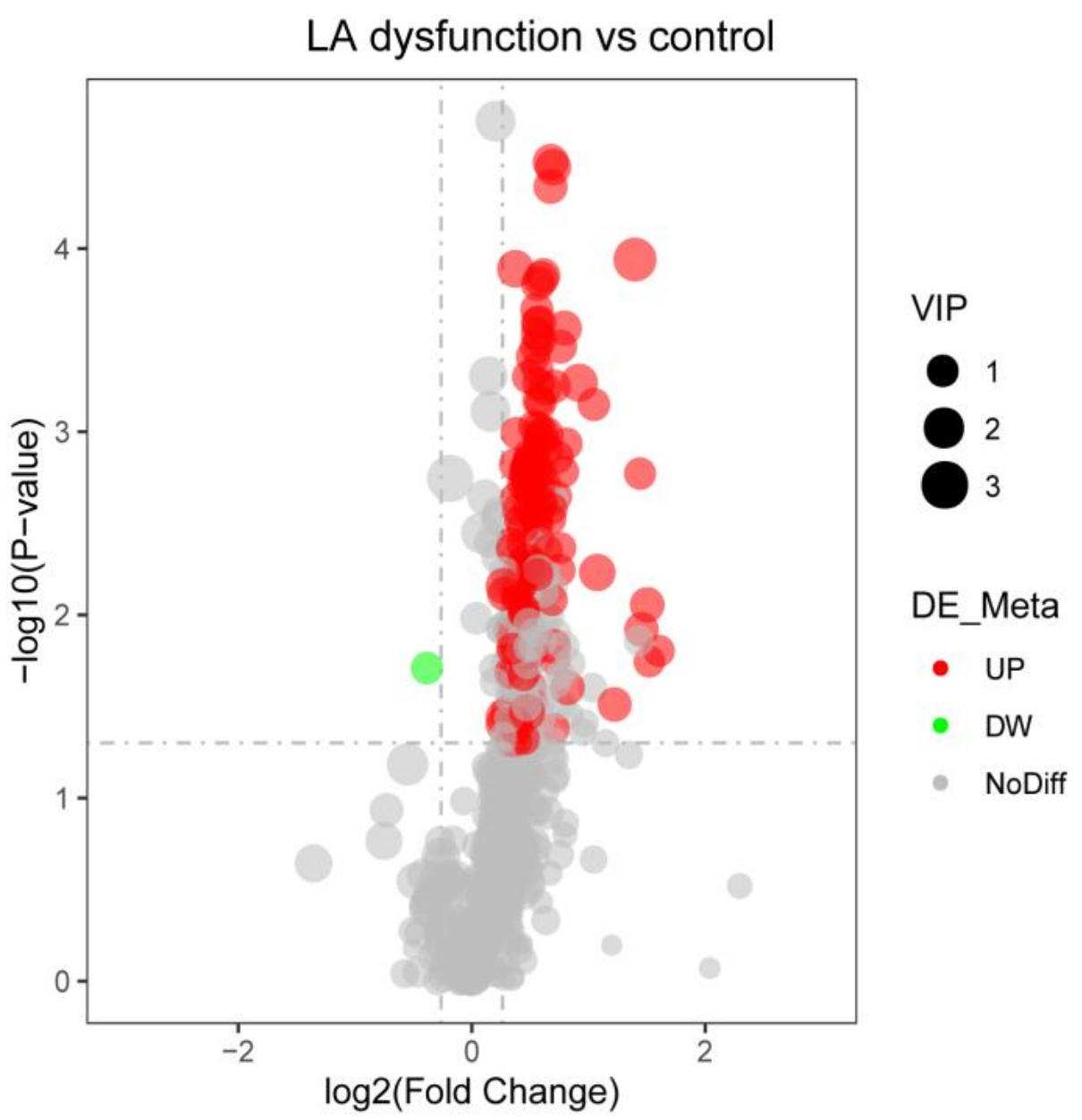

Compared with controls, the LA dysfunction group exhibited 140 upregulated metabolites and 1 downregulated metabolite.

Figure S2. KEGG enrichment bubble plot in patients with LA reserve dysfunction

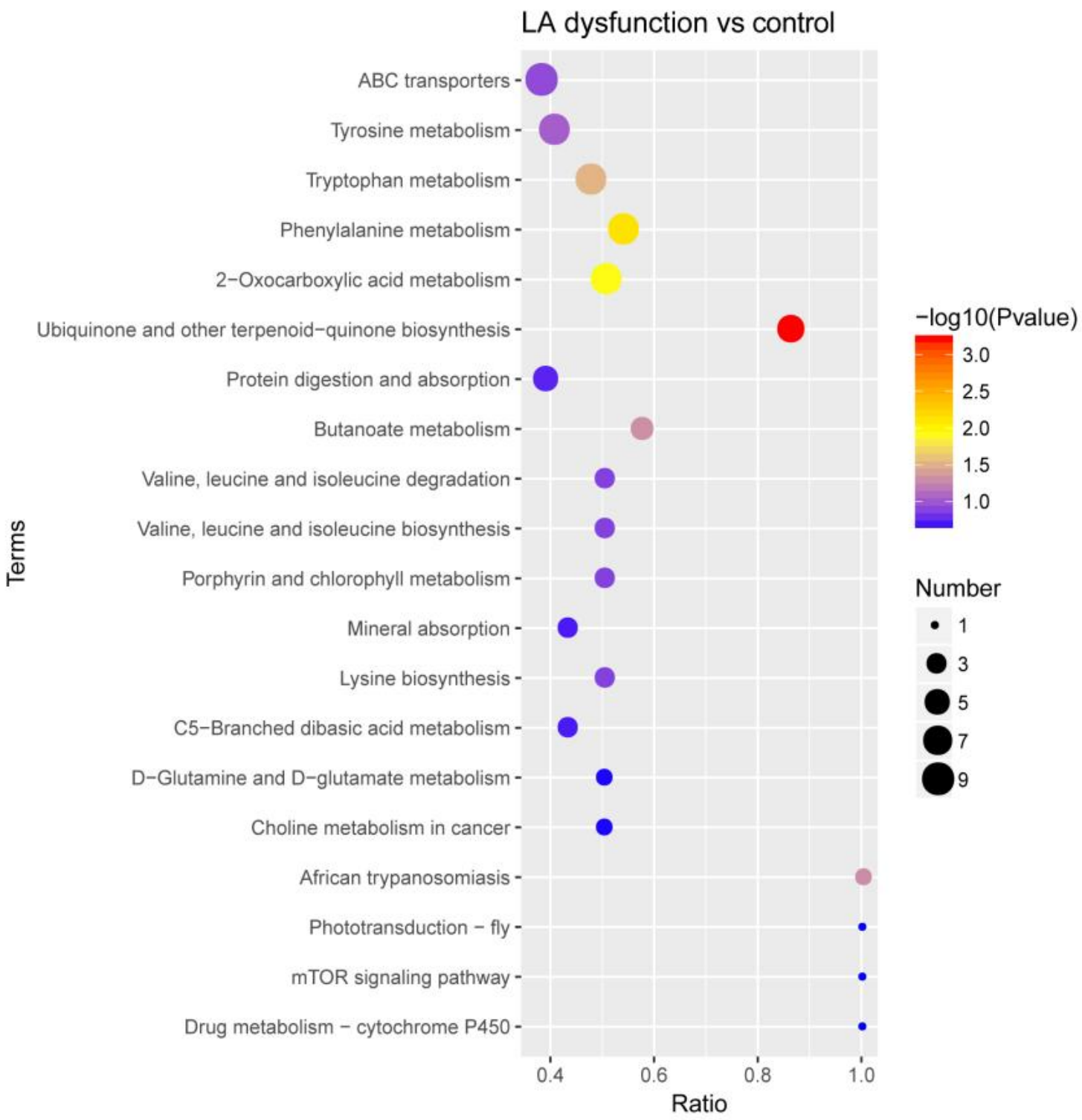

KEGG pathway analysis identified significant associations between early LA dysfunction and ubiquinone and other terpenoid quinone biosynthesis.
